# Passive sampling and benchmarking to rank HOC levels in the aquatic environment

**DOI:** 10.1038/s41598-021-90457-3

**Published:** 2021-05-27

**Authors:** Ian John Allan, Branislav Vrana, Jasperien de Weert, Alfhild Kringstad, Anders Ruus, Guttorm Christensen, Petr Terentjev, Norman Whitaker Green

**Affiliations:** 1 Norwegian Institute for Water Research (NIVA), Gaustadalléen 21, 0349 Oslo, Norway; 2 RECETOX, Masaryk University, Brno, Kamenice 753/5, 625 00 Brno, Czech Republic; 3 Deltares, Delft, The Netherlands; 4 Akvaplan-Niva AS, Fram Centre, Langnes, Postboks 6606, 9296 Tromsø, Norway; 5 Institute of North Industrial Ecology Problems (INEP), Kola Science Centre, Russian Academy of Science, Apatity, Murmansk Region Russia

**Keywords:** Environmental monitoring, Geochemistry, Environmental chemistry

## Abstract

The identification and prioritisation of water bodies presenting elevated levels of anthropogenic chemicals is a key aspect of environmental monitoring programmes. Albeit this is challenging owing to geographical scales, choice of indicator aquatic species used for chemical monitoring, and inherent need for an understanding of contaminant fate and distribution in the environment. Here, we propose an innovative methodology for identifying and ranking water bodies according to their levels of hydrophobic organic contaminants (HOCs) in water. This is based on a unique passive sampling dataset acquired over a 10-year period with silicone rubber exposures in surface water bodies across Europe. We show with these data that, far from point sources of contamination, levels of hexachlorobenzene (HCB) and pentachlorobenzene (PeCB) in water approach equilibrium with atmospheric concentrations near the air/water surface. This results in a relatively constant ratio of their concentrations in the water phase. This, in turn, allows us to (i) identify sites of contamination with either of the two chemicals when the HCB/PeCB ratio deviates from theory and (ii) define benchmark levels of other HOCs in surface water against those of HCB and/or PeCB. For two polychlorinated biphenyls (congener 28 and 52) used as model chemicals, differences in contamination levels between the more contaminated and pristine sites are wider than differences in HCB and PeCB concentrations endorsing the benchmarking procedure.

## Introduction

The chlorinated benzenes hexachlorobenzene (HCB) and pentachlorobenzene (PeCB) are ubiquitous in the environment as result of high historical production volumes and persistence. HCB and PeCB are two closely related chemicals listed as priority substances by the European Water Framework Directive and by the Stockholm Convention (Annex A for HCB). The production and use of these two chemicals is now banned because of their bioaccumulative properties, persistence in the environment and toxicity. Both compounds are characterised by similar hydrophobicity (with logK_ow_ ~ 5–5.5) and Henry volatility (Henry’s law constants (HLC) of 0.015 and 0.014 Pa m^3^ mol^−1^ at 25 °C for PeCB and HCB, respectively)^1^. In the absence of major point sources of emissions to the environment , the repeated exchange between soil, air and water compartments over long periods of time can be expected to contribute to reaching regional/continental (northern hemisphere) equilibrium for these two chemicals. Both substances can be considered as multimedia multiple hoppers where consecutive volatilisation/cold condensation processes have contributed to them being globally distributed, even reaching the Arctic^2^. “Multiple hoppers” differentiate from “single hoppers” (with low volatility and tendency to stick to surfaces) and “swimmers” (with high solubility in water) based on their partitioning properties.

Past uses and emission sources of these two chemicals were radically different. HCB was mainly used as fungicide or formed by-product in the manufacture of pesticides and rubber^1^. Peak production was in the late 1970s and early 1980s; up to 10^4^ tonnes per year. Restrictions on production and uses have been implemented in the period 1960s–1990s. As for HCB, there is no reported current use of PeCB. It was used to decrease the viscosity of PCB-based dielectric fluids, in the production of certain pesticides (e.g. intermediate in the production of quintozene) or found in dye carriers. Contemporary primary emission sources of both chemicals are likely to be mostly as by-products of incomplete combustion and industrial chlorination processes. Originally, the production, use, and emission of the two chemicals were not related (although technical grade HCB contains 1.8% PeCB) and it is not expected that degradation pathways in the environment under oxic conditions include each other as transformation products. Therefore, original sources of these compounds to the environment were not the same. This should lead to concentration ratios that vary widely in the environment depending on the distance from respective point sources.

Considering their physico-chemical properties and that production/use have been banned for over 20–30 years, concentrations in air and water at a regional or hemispheric level can be expected to approach equilibrium. Indeed, air concentrations of HCB (vapour phase) measured in recent years at a hemispheric level do not vary appreciably^3–5^. Their levels in air are generally similar among urban, rural, and remote sites, reflecting the persistence and long-range transport of these chemicals^6^. As an example, the average HCB concentration in air measured in the North Sea region in spring and summer of 2009–2010 was 58 pg m^−3^ and very close to the European median background concentration of 45 pg m^−3^ estimated for 2006^4,5^. It has also been proposed that HCB is close to surface water–air phase equilibrium at sites in the Atlantic, North Atlantic/Arctic marine environment^7,8^. Further away from the European continent, near-equilibrium was also observed for HCB in the Bohai and Yellow Seas, bordering some of China’s agricultural regions in 2012^9^.

While PeCB is a relatively less studied chemical, it was also found to be homogeneously distributed in air of the North Sea^5^ with an average concentration of 13 pg m^−3^. In this study, the comparison of air and water concentrations for PeCB identified net deposition for the German Bight in 2009 but phase equilibrium in 2010^5^. Smaller (and perhaps seasonal) fluctuations on short timescales between net deposition or volatilisation can also be indicative of reaching such phase equilibrium^5^. In case of phase equilibrium, the ratio of fugacity (or chemical activity) *f* of a chemical x in water to air approaches unity:

1 $$ \frac{{f_{w,x} }}{{f_{a,x} }} = \frac{{C_{{w{\text{-}}x}} H_{x} }}{{C_{{a{\text{-}}x}} RT_{a} }} $$

where C_w-x_ and C_a-x_ are freely dissolved and vapour phase concentrations in water and air (mol m^−3^), respectively, *H*_*x*_ is the temperature and salinity dependent Henry’s law constant (Pa m^3^ mol^−1^), *R* is the universal gas constant (Pa m^3^ mol^−1^ K^−1^) and *T*_*a*_ is the air temperature (K). If phase equilibrium is being reached for HCB and PeCB, the relationship between relative concentrations of the two chemicals in air and surface waters can be expressed as:

2 $$ \frac{{C_{{w{\text{-}}HCB}} }}{{C_{{w{\text{-}}PeCB}} }} = \frac{{C_{{a{\text{-}}HCB}} H_{PeCB} }}{{C_{{a{\text{-}}PeCB}} H_{HCB} }} $$

According to Eq. (2), at equilibrium, differences in concentration ratios of the two chemicals in air or water will be directly proportional to differences in their *H*_x_ ratios.

Partition-based passive sampling with polymers such as with silicone rubber (SR) represents an effective tool to assess this C_w-HCB_/C_w-PeCB_ in-situ. This is because the amount of chemicals accumulated in the samplers is proportional to the fugacity in water and the relationship between the two can be quantified with a known uncertainty^10,11^. HOC uptake into passive samplers first follows a linear accumulation when HOC masses absorbed remain far from expected values at equilibrium. During this phase of uptake, masses of contaminants accumulated are proportional to the fugacity in water through a sampling rate R_s_ estimate from the use of performance reference compounds^10–12^. When samplers are left for a sufficiently long period of time in water, HOC uptake plateaus as the chemical activity in the sampler approaches the same chemical activity as in water. At equilibrium, the mass of chemical absorbed in the sampler is proportional to the freely dissolved concentration in water through the sampler-water partition coefficient, K_sw_.

In the present study we hypothesise that, in the absence of active emission sources, if both HCB and PeCB are reaching phase equilibrium at a regional or pan-European scale, the concentration ratio of the two substances (HCB/PeCB) in water should be near constant over large geographical distances. While this ratio is expected to be constant, it does not necessarily mean that absolute freely dissolved concentrations will be. These may vary e.g. as a result of sorption to phases not previously at equilibrium with the water (e.g. during phytoplankton bloom). In a first step, we investigated the European passive air sampling data for C_a-HCB_/C_a-PeCB_ obtained from the Genasis (http://www.genasis.cz) database for the period 2012–2018. We then evaluated how the ratio of freely dissolved concentrations of HCB and PeCB in surface water varies at locations across Europe and the European Arctic. Here for we used a unique set of absorption passive sampling data obtained over a decade at sampling sites located at latitudes between 35 and 79°N, through different monitoring and research activities. Samplers were deployed in freshwaters (rivers, lakes and canals) and/or at marine/coastal sampling sites in the south of Norway, in the Norwegian and Russian Arctic, in the Netherlands and in the Czech Republic, Greece and Portugal. Also included were data from the mobile dynamic passive sampling unit used in the Danube river and in the Black Sea^13^. Based on the outcome, we propose that this HCB/PeCB diagnostic ratio can be used for benchmarking (i) to indicate presence of active or point pollution sources of one of the two chemicals, (ii) for benchmarking of concentrations of other chemicals sampled with silicone rubber with the aim to identify potential presence of pollution sources and compare their levels over large distances. Finally, since both substances are on the list of priority substances of the European Water Framework Directive (WFD), we elaborate on possible implications of these results for chemical water quality monitoring in Europe.

## Methods

In the following section, we provide details of our generic procedures for the preparation, extraction, and analysis of passive samplers. However, over the long period of investigations (2009–2019), method modifications have been implemented as a result changes in analytical instrumentation or improvements. When the analytical methodology was modified (e.g. changing the extraction solvent or transferring instrument analysis for polychlorinated biphenyls (PCBs) and organochlorinated contaminants (OCs) froman electron capture detector to a mass spectrometer), a new method validation was conducted. Since three different laboratories undertook analyses, exact procedures are slightly different for the three laboratories.

### Materials

All glassware was first washed in a professional laboratory dishwasher (Miele, Germany) followed by either solvent rinsing or baking in a muffle furnace at 540 °C. All solvents (dichloromethane, *n*-hexane, methanol, *n*-pentane) were from Rathburn (UK), with the exception of cyclohexane that was from J.T. Baker (USA). All were of high-performance liquid chromatography (HPLC) grade or better. Analytical-grade standards for deuterium-labelled polycyclic aromatic hydrocarbons (PAHs) used as performance reference compounds (PRCs) were from Chiron. Purities were > 99.5% for deuterated PAHs. Analytical standards of investigated compounds and analytical recovery standards for PCBs and OCs were of similar grade and obtained from LGC/Promochem (UK).

### Silicone rubber preparation

For all passive sampler exposures, two formats of silicone rubber passive samplers were used. Samplers were either cut into long strips or small rectangles. Since the study period spans a decade, these sampler exposures were undertaken with samplers made from different batches of the same polymer. AlteSil silicone rubber samplers (with 0.5 mm thick AlteSil sheets purchased from Altecweb, UK) were prepared following procedures used and described previously^14,15^. When silicone rubber samplers were prepared and analysed in other laboratories, procedures followed were similar to those described below and have been reported elsewhere^16^. Polymer sheets were cut to appropriate dimensions (e.g. 100 cm × 2.5 cm wide × 0.5 mm thick) before being cleaned in a Soxhlet extractor using ethyl acetate to remove oligomers from the polymer. The polymer strips were then air-dried to remove the ethyl acetate and placed in a glass jar for further cleaning by partitioning with methanol renewed multiple times. Prior to the spiking of PRCs^17^. For reference, at RECETOX, this procedure is conducted under vacuum to reduce potential for contamination. PRCs are isotopically non-naturally occurring, sometimes labelled-analogues (deuterated PAHs at NIVA, non-occurring PCBs at RECETOX) of chemicals of interest, that can dissipate from the samplers during exposure. The dissipation of PRCs from the samplers during exposure allows us to estimate exchange kinetics during deployment in-situ^12^. PRC spiking involved adding known amounts of deuterated PAHs to a batch of silicone rubber membranes that were soaking in methanol. The methanol solution was regularly supplemented with ultrapure water over time to decrease the solubility of the chemicals in solution, and thereby forcing their distribution to the silicone rubber. This procedure results in homogenous concentrations of PRCs in the silicone rubber as needed for adequate use of the PRCs. Once prepared, all samplers were placed in sealed and clean metal or amber glass with metal lined screw caps containers at − 20 °C until exposure. For most exposures one sample was formed of two strips and this corresponds to having 1000 cm^2^ of sampling surface and a nominal sampler mass of 30 g. In some cases, only one strip (15 g of silicone rubber) was used. For certain studies (e.g. in^16^), a different configuration and set of PRCs was used with equivalent or improved robustness of the measurements.

### Silicone rubber extraction and analysis

After deployment, samplers were retrieved, and their surface washed on-site using water they were deployed in. Back in the laboratory, samplers were further rinsed with ultrapure water and dried with a clean tissue to remove any residual fouling. All samplers, including field and preparation controls, were extracted overnight by soaking with *n*-pentane (2 × 150 mL) with recovery standards added to the extraction jar during the first step of the extraction. The volume of pentane was reduced to 2 mL by exposing the sample to a gentle flow of nitrogen at room temperature. In most cases, extracts were first split into two equal fractions by volume. One fraction received a general clean-up using gel permeation chromatography (GPC, with dichloromethane as mobile phase). This post-GPC sample was reduced in volume (~ 200 µL) under a gentle stream of nitrogen gas and analysed for PAHs/PRCs. The other fraction received treatment with 2 × 1 mL sulfuric acid (H_2_SO_4_), and was reduced in volume before being analysed for PCBs and OCs^18^. The general clean-up and GPC procedures have been described elsewhere^15,19^. Analysis for PRCs and OCs was performed on an Agilent 7890A gas chromatograph coupled to an Agilent 5975c inert XL EI/CI quadrupole mass spectrometer operated in single-ion monitoring mode (SIM) with electron impact ionisation (70 keV)^20,21^. Analyte separation was on a DB-5MS column (30 m, 0.25 mm inside diameter and 0.25 µm film thickness; Agilent JW Scientific) with a 1 µL pulsed split-less injection (pulse pressure 20 psi for 1.2 min and injector temperature of 300 °C). Helium was used as carrier gas with flow set to 1.2 mL min^−1^. The oven temperature program for the GC consisted of a step at 60 °C (held for 2 min) before an increase to 250 °C (at the rate of 7 °C min^−1^) and a final increase to 310 °C (at the rate of 15 °C min^−1^), when this temperature was held constant for a further 5 min. Temperatures for the ion source, quadrupole, and transfer line were 230, 150 and 280 °C, respectively. Quantification was performed using the relative response of surrogate internal standards and 7-point calibration curves. Deviation (< 20%) of the qualifier ion response relative to that of the quantifier ion was used for identification^21^. Recovery standards were naphthalene-d_8_, biphenyl-d_10_, acentphthene-d_8_, dibenzothiophene-d_10_, pyrene-d_10_, benz[a]anthracene-d_12_, and perylene-d_12_ for PAHs and CB30, CB53, and CB204 for PCBs/OCs. Recoveries of target compounds (PAHs and PCBs/OCs) during the pentane extraction of SR passive samplers were in the range 90–110%. For samplers analysed in the Czech Republic and in The Netherlands, we refer to previous work^16,22,23^.

### Passive sampler exposures

Depending on the particular study, passive sampler exposures ranged from 1.5 days (using the mobile dynamic passive sampling unit in the Danube river or in the Black Sea) to months (e.g. 3 monthly exposures in Norwegian rivers during the period 2012–2016). For sampler exposures at particularly remote sampling locations (e.g. Jan Mayen and Bear Island), samplers were left deployed for an entire year. The list of sampling locations (air and water) with their coordinates, exposure dates and durations are given in Tables SI-1 to 5 and 8 in supporting information.

### Estimation of freely dissolved concentrations

Data treatment was the same for all samplers regardless of the sampling dates, exposure duration or water temperature or salinity. No corrections for water temperature or salinity were applied to polymer-water partition coefficients, *K*_sw_ for PRCs or chemicals of interest. Sampling rates, *R*_*s*_ (L d^−1^) were estimated for each sampler at each site by applying the non-linear least square (NLS) method to the PRC dissipation data using the methodology presented by Booij and Smedes^24^. This is done by assuming that *f*, the fraction of PRCs remaining in the sampler after exposure (N_t_/N_0_), is a continuous function of the sampling rate:

3 $$ f = \frac{{N_{t} }}{{N_{0} }} = exp\left( { - \frac{{R_{s} t}}{{mK_{sw} }}} \right) $$

The model to estimate *R*_*s*_ from *K*_sw_ was that given in Rusina et al. for AlteSil SR^25^. Since it relies on the assumption that the uptake in the samplers for most hydrophobic substances is under water boundary layer-control, *R*_*s*_ is proportional to the mass transfer coefficient in the boundary layer. The model is based on the use of the optimised (from the NLS above) exposure specific *β*_*sil*_ factor (L^1.08^ kg^0.08^ d^−1^) and known *K*_sw_ (L kg^−1^):

4 $$ R_{s} = \beta_{sil} K_{sw}^{ - 0.08} $$

The complete equation, taking into account linear, equilibrium and partially equilibrated conditions, was used to estimate dissolved concentrations of HCB, PeCB, and selected substances of interest:

5 $$ C_{w} = \frac{{n_{acc} }}{{K_{sw} m\left( {1 - e^{{\frac{{ - R_{s} t}}{{K_{sw} m}}}} } \right)}} $$

with *C*_w_ being the freely dissolved concentration (ng L^−1^), *n*_acc_ the mass of chemical accumulated in the sampler during exposure, *m* the mass of the SR passive sampler and *K*_sw_ the AlteSil-water partition coefficient measured for.

The degree of equilibrium (DEQ^16^) reached by the sampler for the uptake of HCB in exposures to the Drammen and Glomma rivers was calculated with the following equation:

6 $$ DEQ = \left( {1 - \exp \left( { - \frac{{R_{s} t}}{{K_{sw} m}}} \right)} \right) $$

Once we had established that HCB and PeCB can be used for benchmarking, we needed to select chemicals to which benchmarking could be applied. We chose two polychlorinated biphenyl congeners 2,4,4′-trichlorobiphenyl (CB 28, CAS number 7012-37-5) and 2,2′,5,5′-tetrachlorobiphenyl (CB 52, CAS number 35693-99-3), for which *K*_sw_ are available for AlteSil SR^26^ and for which we had the highest amount of data above LOQ.

### Quality assurance and control

Multiple levels of quality assurance and control were used during this work. All SR exposures (or sets of) included the use of field blanks/controls to evaluate possible contamination as a result of sampler manipulation during deployment and retrieval operations. Preparation blanks/controls were also regularly used to assess contamination during preparation and laboratory storage of the samplers. For each batch of sampler analysis, solvent blanks were used to assess contamination during SR extraction and analysis. Since 2012, we have put in place the use of an in-house reference material to check the performance of the extraction/analytical procedure. A total of 70 SR (nominal mass of 4 g per SR) were homogenously dosed with all chemicals analysed in SR at NIVA on a regular basis following a procedure similar to that used to spike PRCs. In most cases, for each SR batch of analysis, one of these SR sheets was analysed to evaluate the inter-batch variability in the analysis. No trends in HCB and PeCB amounts measured in these samplers could be observed over time. The relative standard deviation for masses of HCB and PeCB (5.1 and 7.9 ng sampler^−1^) measured in these samplers were 11.5 and 11.5%, respectively for 21 batches of analysis. This indicates relatively low inter-batch variability, which is difficult to distinguish from the variability in the original spiking of the chemicals into these QA samplers. Finally, all three laboratories involved in the analysis of SRs in the present work participated to the three QUASIMEME proficiency testing schemes (http://www.quasimeme.org; scheme DE-13) and obtained excellent results for the extraction and analysis of target substances from SR passive samplers (Z-scores mostly < 1) as well as for the calculation procedure described above.

### European air concentrations for HCB and PeCB from the Genasis database

We obtained passive air and gas phase sampling data from the GENASIS database (RECETOX) in June 2018 and the HCB and PeCB passive air concentration data requested was for European background sampling sites for the period 2005–2018. The data for the period 2005–2012 was very incomplete in terms of sampling sites and years covered. We restricted our data use first to the period 2012–2018 as it was most relevant to our passive water sampling dataset and to data from all sites or limited the dataset for sites of the European Monitoring and Evaluation Programme (EMEP) under the Convention on Long-range transboundary Air Pollution (CLRTAP). For the calculation of HCB/PeCB ratios of air concentrations, we removed paired HCB and PeCB data when either of these were below limits of quantification (mostly for PeCB). The proportion of these data in the whole dataset was negligible.

## Results and discussion

### Background passive air concentrations for HCB and PeCB

**Figure 1:**
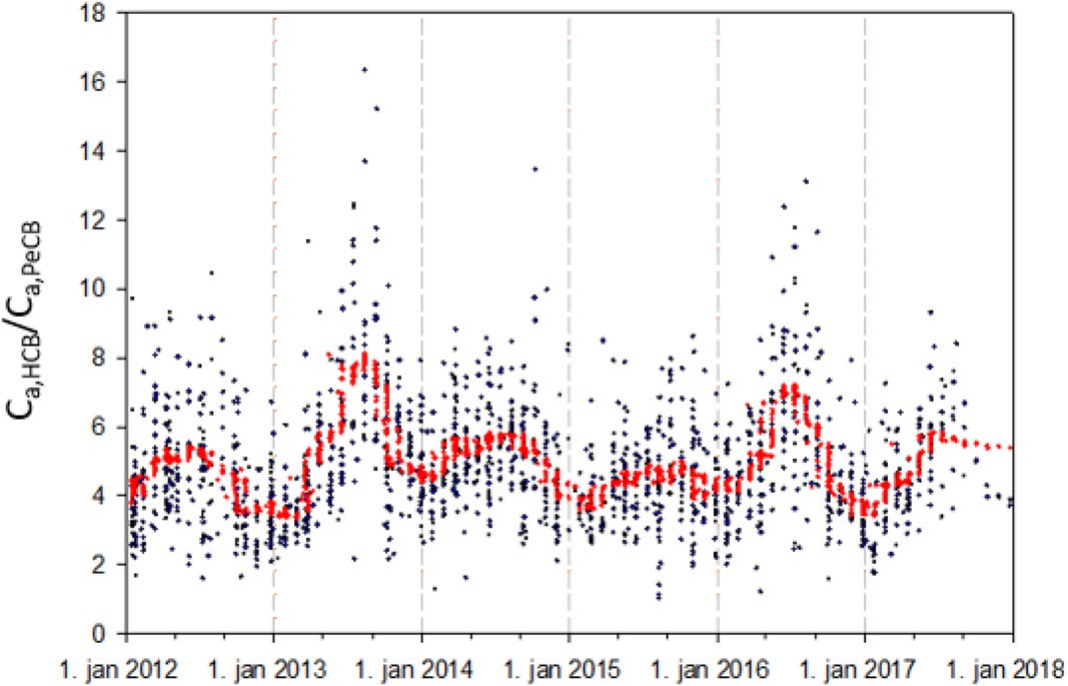
Temporal variations in HCB/PeCB ratios for passive air concentrations measured at European background sampling locations. Note: in red is the 7-day moving average of all data and is given only for visual impression.

The median passive air concentration of HCB and PeCB for the period 2012–2018 (n = 1622) were 67.0 and 14.8 pg m^−3^, respectively. These median concentrations are in excellent agreement with concentration levels reported in previous studies for air concentrations which were not necessarily measured by passive air sampling^5^. The median value of HCB/PeCB concentration ratio was 4.59 based on this dataset. When focusing solely on EMEP monitoring locations, median HCB and PeCB concentrations were 65.6 and 14.6 pg m^−3^, respectively (n = 108). The median of HCB/PeCB ratio was similar to that found for the entire dataset. In Fig. 1, we plotted the ratio of air concentrations for HCB over PeCB for all monitoring stations (2012–2018) as well as a 7-day moving average to facilitate visualisation of the pattern in the data. The HCB/PeCB ratio varies from a minimum value of 1.02 to a maximum of 16.3. For all data combined, regardless of the monitoring station or sampling year or season, the 25th percentile, median and 75th percentile were 3.62, 4.59 and 5.85, respectively. In Fig. 1, a seasonal pattern is also clearly distinguishable with lower HCB/PeCB ratio in the winter and higher ratios in the summer. The median of ratio for all stations for the months of November to February 2012–2018 is 3.98 and 5.30 for the months of May to September. While the pattern is obvious, it is difficult to distinguish whether it is the result of actual relative changes in air concentrations of HCB and PeCB or if it is operational, i.e. because of the measurement itself with e.g. a lack of temperature correction of the passive sampling data or because of a combination of the two. From Eq. (2), the ratio of HCB/PeCB concentrations expected in water in the case where air and water concentrations are close to equilibrium can be estimated. Dimensionless Henry’s law constants reported for HCB and PeCB are very similar (0.015 and 0.014, respectively at 20 °C) and expected change in H with change in temperature (dlnH/d(1/T) of 6000 and 5200 for HCB and PeCB, respectively) for the two compounds are relatively similar^27^. Based on these values, the ratio of H_PeCB_/H_HCB_ can be expected to vary from 0.76 at 0 °C to 1.02 at 30 °C (Table SI-6 and Figure SI-1). A correction for the difference in molecular weight for the two compounds (250.3 and 284.8 g mol^−1^) amounts to a factor of 1.13. According to Eq. (2), the expected C_w-HCB_/C_w-PeCB_ ratio from the median C_a-HCB_/C_a-PeCB_ ratio of 4.59 would be in the range 3.70–4.28. These values do not include any correction of H values for water salinity. At this point, it may also be useful to indicate an approximate range of ratios outside of which values can be considered as outliers. Indicative limits to identify outlier ratios in water were calculated from air ratios at EMEP sites. The median value and first and third quartiles were estimated. The interquartile range (IQR, i.e. difference between the first and third quartile) was used to estimate the upper limit. By adding 1.5 × IQR to the third quartile, we obtain a value of 7.9. Since subtracting 1.5 × IQR from the first quartile results in a negative ratio, we inverted the HCB/PeCB ratio into a PeCB/HCB ratio and estimated the upper limit in the same way as for the original ratio. Back-transforming to an HCB/PeCB ratio results in a lower limit of 2.4. Indicatively, corresponding limits for the identification of outliers are 2.2 and 7.0 for water based on the H_PeCB_/H_HCB_ ratio at 15 °C. This range is given for information only and will require further refining with a more appropriate dataset. However, values that fall outside this range warrant further investigation for possible contamination with one of these compounds.

### HCB/PeCB ratio in the Drammen and Glomma rivers for the period 2013–2016

The rivers Drammen and Glomma, which are at a similar latitude in the South of Norway, were sampled on a continuous basis with consecutive SR exposures four times a year over a three-year period. There are no known specific sources of HCB or PeCB in these two rivers fed by water from large lakes. We can therefore assume concentrations for these two chemicals are the result of long-range transport or exchange with the air. Duplicate SR measurements were undertaken with sampling rates R_s_ spanning over an order of magnitude (from < 1 L d^−1^ to 50 L d^−1^) and with strong differences in degree of equilibrium (DEq) reached. This is the result of the samplers exposed in winter or summer (DEq from < 0.1 to 0.9 for a model compound with log*K*_sw_ = 4.5) with large differences in deployment temperature in water (0–20 °C). Despite these large differences, C_w-HCB_/C_w-PeCB_ ratio does not vary appreciably in both rivers, demonstrating a high correlation of freely dissolved HCB and PeCB concentrations in water calculated from passive sampling in both rivers (Figure SI-2). Remarkably, the HCB/PeCB ratios were extremely similar for both rivers. Linear regressions were C_w-HCB_ = 3.93 (se = 0.24; *p* < 0.0001) × C_w-PeCB_ with R^2^ = 0.930 for the river Drammen and C_w-HCB_ = 4.11 (se = 0.21; P < 0.0001) × C_w-PeCB_ with an R^2^ = 0.96 for the Glomma. The slopes (or C_w-HCB_/C_w-PeCB_ ratio) of the regressions for the two rivers had very low standard errors and fall well within the range of 3.70–4.28 expected from European air data. These results confirm air and water concentrations of HCB and PeCB are strongly connected in these two rivers.

At first, it may be surprising that relative concentrations of HCB and PeCB are so consistent despite the order of magnitude-range in estimated C_w_ (Table SI-8). However, in these two rivers, the passive sampling measurement based on sampling the freely dissolved HOCs in water is close to measuring their total concentration. Considering the reported levels of SPM, SPM-associated concentrations of HCB (< 0.3–1 ng g^−1^ dry weight SPM) and PeCB (< 0.3 ng g^−1^ dry weight SPM), dissolved organic carbon (DOC) and literature-based K_DOC_ (i.e. log*K*_DOC_ of 4.4 for HCB from^28^), sorbed concentrations of HCB and PeCB represent only a minor proportion (< 10% for HCB and less for PeCB) of the total concentration in water (SI section, Table SI-7). Additional sorption to SPM, plankton or DOC at the levels of a few mg L^−1^ will not affect the dissolved concentrations to any extent at these conditions (< 10%). The amount of organic carbon in the water needs to be tens of mg L^−1^ for the relative phase distribution of HCB to change substantially. This confirms that the observed constancy of the HCB/PeCB ratios in these two rivers is genuine. On a short-term basis, degradation processes are not likely to play a more significant role in the losses of one compound over the other since both compounds have been shown to have long half-lives in the environment^1^. The variability in the C_w-HCB_/C_w-PeCB_ ratio for these two rivers (Figure SI-3) is equivalent to C_w-HCB_ and C_w-PeCB_ varying randomly by 15–20% around a mean C_w_ value resulting in a ratio of 4.1.

### HCB/PeCB ratio in other freshwater environments

**Figure 2:**
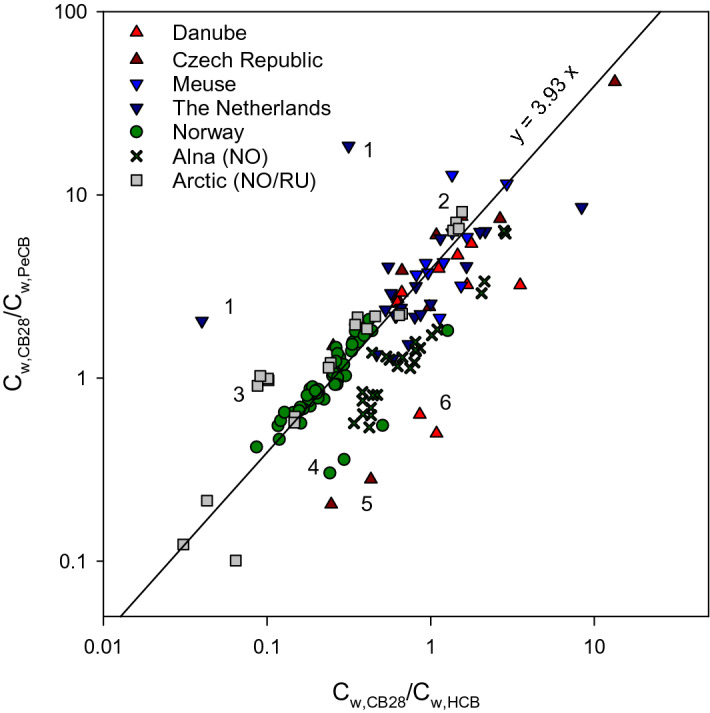
Benchmarking of freely dissolved concentrations of CB28 with those estimated for HCB and PeCB for all freshwater sampling sites. Note that every datapoint is plotted on the graph. Sites closest to the top right corner exhibit highest CB28 concentrations while those closest to the bottom left corner indicate lowest CB28 concentrations. Datapoints deviating from the reference diagonal line indicate either a PeCB contamination (towards the top left corner) or an HCB contamination (towards the bottom right corner). 1: Canals in The Netherlands with HCB contamination; 2: PCB contamination in the Pasvik river; 3: HCB contamination of the Pechenga river; 4 and 5: Low C_w-HCB_/C_w-PeCB_ for the Rivers Alna and Borgebekken in Norway and Morava River in the Czech Republic; 6: PeCB contamination in the Danube River.

The next step was to assess how C_w-HCB_/C_w-PeCB_ ratios varies when extending the evaluation to freshwater sites over a larger regional scale. The data are summarised in Fig. 2 and shown in detail in Figure SI-5. Figure 2 presents two levels of information: (i) possible local contamination with either HCB or PeCB indicated by a deviation from the diagonal reference line (slope equivalent to a HCB/PeCB ratio of 3.93), and (ii) benchmarking of freely dissolved concentrations of CB28 with those for HCB and PeCB measured at all freshwater sampling locations along the reference line. The gradient of CB28 contamination however, will be discussed later. In Fig. 2 and Figure SI-7, we show that a majority of freshwater sites follow the reference line equivalent to a C_w-HCB_/C_w-PeCB_ ratio of 3.93. Most of the datapoints from large rivers, such as the Meuse and Danube rivers, are in line with ratios obtained for the Glomma and Drammen rivers. Some notable outliers indicating HCB contamination include two canal or brook sites in The Netherlands (noted 1 on Fig. 2) and the Pechenga and Titovka rivers (noted 3 on Fig. 2) in the Kola Peninsula. HCB/PeCB ratios for these sites are a factor of 2–3 or more above the slope of Fig. 2. Sites with lower ratios (below the reference line) appearing to indicate PeCB contamination include the Alna river in Oslo, Borgebekken in the South of Norway (noted 4 on Fig. 2) as well as selected locations on the Morava in the Czech Republic (noted 5 on Fig. 2) on the Danube (noted 6 on Fig. 2) or the Meuse rivers (“Getijden Maas Boven”).

The boxplot of Figure SI-5 shows the range of C_w-HCB_/C_w-PeCB_ found for riverine sites listed according to latitude from Norway or the Russian Arctic, The Netherlands, as well as in the Czech Republic and countries that the Danube flows through sampled during the period 2010–2019. Rivers shown in blue are sites that are not expected to exhibit contamination with either of the two chemicals and are therefore representative of background levels (absence of industry, low population density or urban environments). As expected, for most of these sites, the HCB/PeCB ratio is close to the ratio of 4.2 mentioned above and within the indicative outlier range of 2.2–7.0. For the stream Borgebekken and the river Alna, a consistently lower C_w-HCB_/C_w-PeCB_ ratio was found. Both flow through industrial areas and have been the subject of past contamination with a range of HOCs. The river Lysaker close to Oslo (Norway), despite being in urban settings, does not show any appreciable deviation from the expected ratio. This is also the case for the Pasvik river and lake Salmijarvi with sampling sites mostly downstream of the Russian town of Nikel in the Arctic, site of a nickel smelter. At these locations no increased levels of PeCB relative to HCB caused by past contamination were found. A ratio consistently between 5 and 6 was observed for the Kola river upstream of Murmansk (Russia) also does not appear to stand out. All sites from the Tana to the Kola rivers are on a similar latitude. These rivers should exhibit similar C_w-HCB_/C_w-PeCB_ ratios if the chemicals in water are at background levels. This would be the result of long-range transport or dictated by air concentrations in the absence of local or regional sources of contamination. Two sites on the rivers Pechenga and Titovka sampled on multiple occasions consistently stand out with ratios between 8 and 11, likely caused by a relative increase in concentrations of HCB. The data from The Netherlands, such as from the river Meuse, are mostly consistent with theory. A lower ratio can be observed for a few sites including one sampling event on the Meuse. A number of sites exhibit outstandingly high HCB/PeCB ratios in The Netherlands including one site on the river Meuse as well as smaller brooks and canals (Afleiding canal) and particularly the Apeldoorns canal and Baakse brook with ratios reaching 50–60. For the central European rivers, a wider range of ratios can be observed. HCB/PeCB ratios for the Morava and Svratka rivers are variable but do not stand out ostensibly. Data for the Svitava is mostly in the range of 5–10 indicating relative increase in HCB concentration for this river. The data from the Danube will be discussed in the following section.

### Spatial variation in HCB/PeCB ratio in the Danube

**Figure 3:**
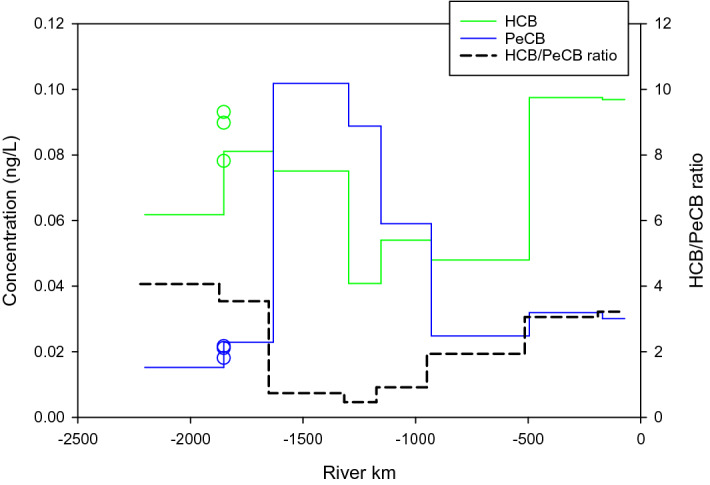
Spatial variation in freely dissolved aqueous HCB and PeCB concentrations and in HCB/PeCB concentration ratio observed from the source of the Danube river to its mouth (km 0) during the Joint Danube Survey 3 (13.08.2013–25.09.2013). Note that the profile for the HCB/PeCB ratio was shifted to the right for a visual improvement.

The successive sampling of different stretches of the Danube over 60 days in 2013 during the Joint Danube Survey 3 (see Table SI-3) provides us with a very detailed picture of relative changes in freely dissolved concentrations of HCB and PeCB over the entire course of the river^16^. The HCB/PeCB ratio in the upper sections of the Danube (1700 km or more upstream from the delta) is as can be expected as a reflection of background conditions, close to 4 (Fig. 3). It then drops to close to 1 before gradually increasing again to 3 when reaching the delta in Romania. More conventional stationary deployments of passive samplers near Bratislava (shown as circles on Fig. 3) confirmed the ratio of 4 for the upper part of the river and thereby validating the sampling methodology put in place for sampling the Danube^16^. As shown on Fig. 3, the drop in HCB/PeCB ratio from 4 to 1 clearly results from the increase in concentration of PeCB. This generally confirms our assumption that a decrease of this ratio tends to indicate relative higher PeCB concentrations and vice versa. The increase in C_w-PeCB_ likely takes its origin in the river Sió, a right bank tributary of the Danube in Hungary. The confluence with the Danube is at the river km 1497. Highest PeCB concentrations in sediments of the whole Danube were found for this point^29^. It is difficult to trace the exact source of PeCB, mainly because the Sió drains more than one third of Hungarian Transdanubia (river basin close to 15,000 km^2^). The impact of the load from the Sió can be suspected as a main source of PeCB concentration in the sediment of the Danube downstream of the Sió confluence.

### The HCB/PeCB ratios in the marine environment

**Figure 4:**
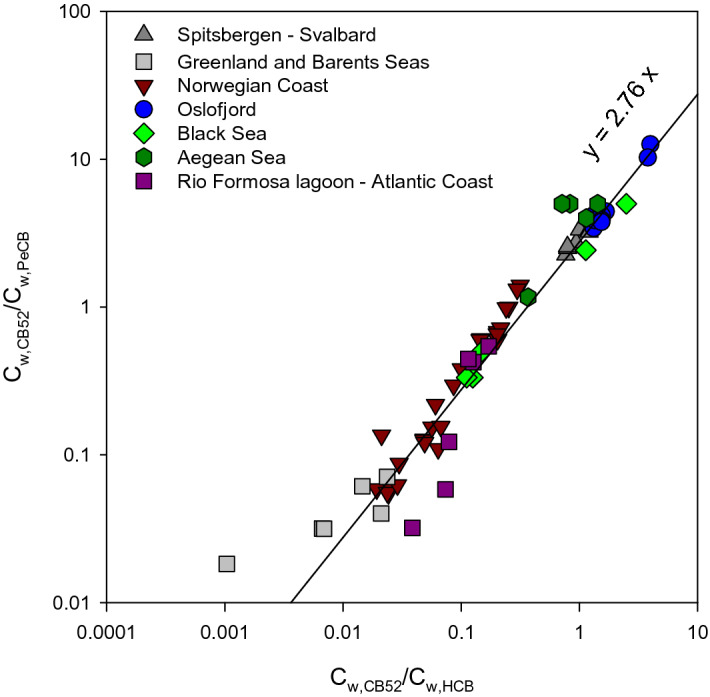
Benchmarking of freely dissolved concentrations of CB52 with those estimated for HCB and PeCB at all marine sampling sites. Note that every datapoint is plotted on the graph. Sites closest to the top right corner exhibit highest CB52 concentrations while those closest to the bottom left corner indicate lowest CB52 concentrations. Datapoints deviating from the diagonal reference line indicate either a PeCB contamination (towards the top left corner) or an HCB contamination (towards the bottom right corner).

With significantly larger surface area for air–water exchange and volumes of water, such constancy in C_w-HCB_/C_w-PeCB_ ratios should also be observed in the marine environment at a regional/continental scale in agreement with previous research indicating near phase equilibrium conditions at certain marine locations. Data acquired over the period 2009–2019 with SR for a range of marine or coastal sampling locations from the Aegean Sea to the Arctic are presented in Fig. 4. Figure 4 displays, similar to Fig. 2 benchmarking of freely dissolved concentrations of PCB congener 52 over those for HCB and PeCB for all marine sites. While benchmarking ratios vary over the same magnitude as for freshwater sites, most datapoints sit much closer to the reference line indicating very similar HCB/PeCB ratios for all marine sites. These data are presented in detail in a boxplot in supporting information (Figure SI-6). Values of C_w-HCB_/C_w-PeCB_ are for most of the sampling locations under study are between 2 and 4. More precisely, sites shown in blue in Figure SI-6 exhibit very similar ratios. These sites include yearly deployments in extremely remote areas of the Greenland, Barents Seas, and Svalbard. Sampling sites in the Oslofjord and at Hvaler (Skaggerak) are also not expected to be influenced by major point source contamination. The two coastal sampling sites near Kristiansand (Norway), Svensholmen and Glencore quay do not show appreciable differences. The C_w-HCB_/C_w-PeCB_ ratio observed for the inner Oslofjord is slightly lower than the data obtained for yearly deployments further out in the fjord. While not conclusive, this may be connected to the relatively higher levels of PeCB observed in the Alna river (Alna datapoints in Fig. 3) that flows into the inner Oslofjord and the low water exchange rate for inner Oslofjord water. The ratio obtained from coastal monitoring near the town of Ålesund on the Norwegian coast is higher and close to 4. The Aegean and Black Sea boxplots, although assembled under one heading, include different sampling locations. The median ratio of each boxplot is close to the ratio observed for sites not under the impact of contamination with any of the two chemicals. However, in both cases, sampling was conducted at sites with either increased concentrations of HCB for the Aegean Sea or PeCB for the Black Sea.

As shown on Fig. 4 and Figure SI-7, there is an excellent correlation between C_w-HCB_ and C_w-PeCB_ for marine sampling locations. The regression of C_w,HCB_ on C_w,PeCB_ has a slope of 2.76, an intercept close to zero (standard error of the slope = 0.06; R^2^ = 0.978, n = 66). Despite the calculated C_w-HCB_ and C_w-PeCB_ spanning over two order of magnitude depending on the sampling location, the HCB/PeCB ratio remains very constant.

This ratio of 2.76, at what can be assumed to be background level is somewhat lower than that observed in freshwater environments. Although we cannot be sure the reason for this difference, we can propose certain processes that may contribute to lower this ratio. If we assume that for background levels, both chemicals are close to phase equilibrium for the air and water compartments, and that the decrease in solubility of HCB with increasing salinity is stronger than for PeCB. This is likely since, despite PeCB and HCB having relatively similar hydrophobicity, PeCB is much more soluble than HCB in water^27^. If the chemical activity (C_w_/S_w_ with S_w_ the solubility of the sub-cooled liquid) of the two chemicals in water are tending to approach that in air, an increase in salinity will have the effect to decrease the solubility S_w_ in water and in C_w_. A more pronounced effect of the salting-out effect for HCB (ΔS_w-HCB_/ΔS_w-PeCB_ > 1) would accentuate this decrease for HCB relative to PeCB and lower the apparent C_w-HCB_/C_w-PeCB_ ratio:

7 $$ \frac{{\Delta C_{{w{\text{-}}HCB}} }}{{\Delta C_{{w{\text{-}}PeCB}} }} = cst \cdot \frac{{\Delta S_{{w{\text{-}}HCB}} }}{{\Delta S_{{w{\text{-}}PeCB}} }} $$

This salting out effect can also affect silicone rubber-water partition coefficients, *K*_sw_ and passive sampling data in general, particularly as we did not attempt any salinity correction to *K*_sw_ values for HCB, PeCB or the PRCs. Sorption of these compounds to phytoplankton in the water column is possible and would tend to reduce dissolved concentrations. While it would be surprising that kinetics of uptake of PeCB and HCB into phytoplankton would be substantially different, it is plausible that at equilibrium, more HCB would be sorbed to phytoplankton. This would in turn lower HCB concentrations more and lower the ratio. Nonetheless, this difference in HCB/PeCB ratios for marine waters agrees with the assumption that the activity of these chemicals in air dictates activity in water since both ratios are within the range determined to identify outliers.

## Benchmarking PCB concentrations at marine sites

We have established that HCB and PeCB concentrations are at background level for many of the marine sites studied here with no apparent influence from point source of contamination for any of the two chemicals. These HCB and PeCB concentrations in water are likely close to equilibrium with air concentrations on a regional scale. In the case air concentrations are at background levels and homogenous at a regional scale, we propose to benchmark levels of other chemicals in the aquatic environment sampled with SR against C_w-HCB_ and C_w-PeCB_. This procedure was applied to two model chemicals, PCB congeners 28 and 52 and results of the benchmarking can be observed along the reference line of Fig. 4. The main reason for selection was that these were analysed together with HCB and PeCB and were the congeners with the highest frequency of detection in our SR dataset.

Benchmarking of concentrations of CB52 over those of HCB or PeCB are shown in Fig. 4 and in detail in Figures SI-8. While the difference between the lowest and highest observed CB28/HCB and CB28/PeCB ratios is over a factor of 80 (based solely on data above limits of quantification, see supporting information), for CB52/HCB and CB52/PeCB ratios, this difference is over a factor of 250. At first sight, some sampling sites clearly stand out following this benchmarking. These are selected sites in the Aegean Sea, Black Sea and in the Oslofjord. As can be expected, sites with the lowest ratios are the Greenland Sea (Jan Mayen), Barents Sea (Bear Island) and Norwegian Sea (Andøya). These are representative of background levels for CB52. For Jan Mayen, Bear Island, and the Portuguese coast (Faro) specifically, both congeners were below limits of quantification. Some sites such as in the Skaggerak (Hvaler) or in the Norwegian Sea (Ålesund) exhibit levels slightly above background levels seen at other sites which may be the result of anthropogenic emissions from urban areas or land-based contamination input. Higher CB52 levels can be seen for the three Svalbard sites near Kongsfjorden and the settlement of Ny Ålesund.

The higher levels of PCBs identified for the (inner) Oslofjord are not unexpected and are strongly supported by Norwegian coastal water monitoring with Atlantic cod (*Gadus morhua*) which has previously shown elevated PCB levels for the inner fjord (Figure SI-10 and^30^). PCB levels in cod from the Oslofjord are generally an order of magnitude, or more, higher than at other stations on the Norwegian coast. For these marine sampling sites, the procedure appears to function irrespective of whether HCB or PeCB is used for benchmarking. This would tend to indicate that the deviations of the HCB/PeCB ratio from the expected value at background conditions are relatively minor in comparison with differences in PCB concentrations at the different marine sites.

## Benchmarking PCB concentrations at freshwater sites

The application of the benchmarking procedure to freshwater sampling sites is shown along the reference line of Fig. 2 for CB28. This procedure generally distinguishes Czech and Dutch sampling locations from Norwegian and Arctic freshwater sites with lower levels of CB28. Although the basis for sampling site selection for this comparison is opportunistic rather than intentional, these results are as expected when considering differences in levels of urbanisation, population densities and industrialisation between the two regions. Repeated sampling in the Alna river at the same site over a 3-year period shows that benchmarking ratios can vary by over an order of magnitude. The nine sampling events along the Danube continuum shows that CB28 levels can also vary by almost an order of magnitude. Looking at a more detailed picture (Figure SI-9), sites on the Danube, the Morava, Meuse and Svitava rivers stand out and exhibit high ratios for both congeners and benchmarking with both HCB and PeCB. In the Arctic, lake Salmijarvi at the border between Norway and Russia also exhibit high ratios. At a similar latitude, the lowest ratios are found for the rivers Tana, Neiden and Grense Jakob in Finnmark or the Titovka river in the Kola peninsula. This is not surprising since these rivers are relatively remote with low level of urbanisation and industrial development. PCBs present in these rivers are the result of long-range air transport to their respective river basin. The Pasvik river is drains through the Salmijarvi lake and this can explain some of the exposures in the Pasvik river showing elevated benchmarking ratios. Benchmarking against the PeCB concentration also helped identify slightly elevated levels of the two PCB congeners in the Pechenga river. This cannot be observed with the HCB, most likely because of this river is contaminated with HCB (Figure SI-9). Further east, elevated levels of PCBs can also be seen in the Kola river. Further south in Norway, the passive sampling data acquired over a 3-year period, with quarterly exposures of SR in three rivers, tend to show that levels of PCBs in the Alna are higher than in the rivers Drammen and Glomma. This is expected since the Alna is a small urban river that drains into the inner Oslofjord with significant PCB loads and documented previous contamination of sediments with PCBs^31^. Similarly, benchmarking identified elevated PCB levels in the Lysaker river, another urban river that drains into the inner Oslofjord. A paired *t* test with the Drammen and Glomma dataset indicated that ratios of CB28 and CB52 concentrations over those of HCB and PeCB were significantly higher for the river Drammen than for the Glomma (P < 0.01). While this was facilitated by the consecutive SR exposure periods, it also demonstrates the robustness of the passive sampling measurement. The monitoring of other Norwegian rivers and streams, i.e. Toknesbekken, Sandeelva, Grennesbekken and Herlandselva shows benchmarking ratios similar to those obtained for the rivers Drammen and Glomma. Considering the low HCB/PeCB ratios, possibly indicating an active source of PeCB (Fig. 2) for Borgebekken, a specific appraisal of benchmarking with HCB tends to show higher levels of CB28.

Admittedly, the comparison of intrinsic C_w_ estimates for CB28 and CB52 between the different sites provides a very similar spatial distribution of level of contamination at the different sites (Figures SI-11 and SI-12). The ability to obtain this spatial trend in contamination levels, which is built from either C_w_ estimated through Eqs. (3)–(5) or benchmarking based on masses absorbed under linear uptake (Eq. 8 below), shows the robustness of the passive sampling measurement and C_W_ calculation procedure. Indeed, this comparison could be done on a contaminant masses accumulated (n_acc_) without the need to recalculate C_W_ so long as sampling remained linear during the period of exposure *t*:

8 $$ \frac{{C_{{w{\text{-}}CB28}} }}{{C_{{w{\text{-}}HCB}} }} = \frac{{\frac{{n_{{acc{\text{-}}CB28}} }}{{R_{{s{\text{-}}CB28}} t}}}}{{\frac{{n_{{acc{\text{-}}HCB}} }}{{R_{{s{\text{-}}HCB}} t}}}} \approx \frac{{n_{{acc{\text{-}}CB28}} }}{{n_{{acc{\text{-}}HCB}} }} $$

Our study could have been based on a comparison of mass accumulated. However, the application of the ratio of C_w_ instead of n_acc_ allows corrections for cases when passive sampler uptake was not linear. The ratio may deviate slightly owing to the small differences in R_s_ values for the different compounds (see Eqs. 3 and 4). A ranking of contamination levels at different sites could then be done on a basis of chemical masses accumulated in samplers. Crucially, this mass ratio under linear uptake is proportional to the ratio of fugacities of the two chemicals in water.

## Proposal for combined passive sampling-benchmarking procedure

One crucial objective of chemical monitoring programmes is the ability to identify and distinguish sites with elevated HOC levels from other sites. Considering the simplicity of this benchmarking procedure, it could easily be applied at a continental level in order to identify benchmarks through monitoring programmes in connection with the Water Framework Directive or to review data from regional or global monitoring programmes such as AquaGAPS^32,33^. This would enable us to compare relative levels of chemicals at different sites in the aquatic environment independent of factors that influence the passive sampling processes (e.g. differences in R_s_, water temperature, fouling, etc.).

In general, 10–30 g of SR exposed for 1–3 months is sufficient to accumulate enough chemicals from ambient water for analysis. The SR membrane thickness (and exposure time) can be selected to ensure that sampling is in the linear phase of uptake for compounds with logK_pw_ > 4–4.5. A calculation of C_w_ from masses accumulated and sampling rate obtained from PRC dissipation data^24,25^ should be preferred prior to evaluating the HCB/PeCB ratios. However, if HCB, PeCB, and other HOCs of interest are sampled in the linear regime of uptake, masses accumulated can directly be used instead of estimated C_w_ since under these conditions, masses accumulated are proportional to the concentration or activity in water (Eq. 8). The configuration of SR passive samplers can be optimised to ensure linear uptake for chemicals with logK_pw_ > 4–4.5 under most deployment conditions. This can be done by increasing the thickness/volume for a given surface area of the sampler or selecting a polymer exhibiting higher K_sw_ values for chemicals of interest. Ideally, the degree of equilibrium achieved during sampling can and should be checked with the dissipation of PRCs, or with simultaneous deployment of two or more samplers made of the same polymer but with two or more surface to volume ratios^34^. The next step is to calculate the C_w-HCB_/C_w-PeCB_ or n_acc-HCB_/n_acc-PeCB_ ratios for comparison with HCB/PeCB ratios representative of background conditions for fresh or sea water environments. A HCB/PeCB ratios outside the 2.2–7 range for freshwaters tend to stand out and may be the result of elevated levels of either PeCB or HCB. For marine sampling sites, we can be more accurate since the variability in the HCB/PeCB ratio is low and generally is not associated with contaminated sites. In the case of normally distributed data, the interquartile range (IQR) will extend 1.35 × the standard deviation. Outliers are expected at 3 × below the first quartile or 3 × above the third quartile. With this procedure, HCB/PeCB ratios outside the range 2.44–3.08 would indicate possible HCB or PeCB local or regional contamination in the marine environment.

A remaining question is which region can we apply this procedure to. According to our dataset it is likely that we can apply this procedure across mainland Europe from the Black Sea, and north to the Norwegian Arctic and Kola peninsula. In case a gradient of concentration exists from higher concentrations in mainland Europe towards lower concentrations in the Arctic or remote oceanic sites, it is possible that the HCB/PeCB ratios will be relatively constant, though with lower intrinsic concentrations.

## Supplementary Information

Supplementary Information.
