# Supplementary material for: Passive sampling and benchmarking to rank HOC levels in the aquatic environment
Source: Sci Rep. 2021 May 27;11:11231. doi: 10.1038/s41598-021-90457-3 (PMC8159932; doi:10.1038/s41598-021-90457-3)
Supplement: Supplementary file 1 — Supplementary Information. [file 41598_2021_90457_MOESM1_ESM.doc]

**Passive sampling and benchmarking to rank HOC levels in the aquatic environment**

**Ian John Allan^*†^, Branislav Vrana**^ƒ^**, Jasperien de Weert^ξ^, Alfhild Kringstad^†^, Anders Ruus^†^, Guttorm Christensen^‡^, Petr Terentjev^⯍^, and Norman Whitaker Green^†^**

**^†^**Norwegian Institute for Water Research (NIVA), Gaustadalléen 21, Oslo Centre for Interdisciplinary Environmental and Social Research, NO-0349 Oslo, Norway

^ƒ^RECETOX, Masaryk University, Brno, Kamenice 753/5, 625 00 Brno, Czech Republic

**^ξ^**Deltares, The Netherlands

**^‡^**Akvaplan-niva AS, Fram centre, Postboks 6606 Langnes, NO-9296 Tromsø, Norway

**^⯍^**Institute of North Industrial Ecology Problems (INEP), Kola Science Centre, Russian Academy of Science, Apatity, Murmansk Region, Russia

**SUPPORTING INFORMATION**

**1. Monitoring stations and passive water sampler deployment locations.**

The five tables below list the air monitoring locations fromGenasis database[[1]](#footnote-2) silicone rubber sampling sites at freshwater sites and marine locations.

Table SI-1. List of air monitoring locations from the Genasis database (RECETOX, University of Masaryk, Czech Republic) for which data were used in the present study.

| Air sampling location | Coordinates | |
| --- | --- | --- |
|  | latitude | longtitude |
| Aspvreten, EMEP | 58.8058 | 17.38840 |
| Ayia Marina, EMEP | 35.038055 | 33.05778 |
| Bily Kriz, Beskydy | 49.50261 | 18.53856 |
| Birkenes, EMEP | 58.383335 | 8.25000 |
| Brno, Lisen, CHMI station | 49.213215 | 16.67796 |
| Churanov, Sumava, EMEP | 49.06844 | 13.61488 |
| De Zilk, EMEP | 52.29657 | 4.51086 |
| Decinsky Sneznik, Labske piskovce | 50.78951 | 14.08684 |
| Diabla Gora, EMEP | 54.124866 | 22.03808 |
| Giordan lighthouse, EMEP | 36.073334 | 14.21917 |
| High Muffles, EMEP | 54.33494 | -0.80855 |
| Ispra, EMEP | 45.816666 | 8.63333 |
| Jesenik, Jeseniky | 50.24225 | 17.19022 |
| Karvatn, EMEP | 62.783333 | 8.88333 |
| Kosetice, EMEP station | 49.57345 | 15.08041 |
| La Coulonche, EMEP | 48.644993 | -0.45833 |
| Lahemaa, EMEP | 59.515278 | 25.92806 |
| Le Montfranc, EMEP | 45.809994 | 2.06000 |
| Leova, EMEP | 46.5 | 28.30000 |
| Mace Head, EMEP | 53.33 | -9.90000 |
| Mikulov, Sedlec | 48.79175 | 16.72450 |
| Most | 50.510334 | 13.64522 |
| Moussala, EMEP | 42.17916 | 23.58528 |
| Napajedla, Komenskeho str. | 49.170555 | 17.51632 |
| Pallas, EMEP | 68.00048 | 24.24566 |
| Payerne, EMEP | 46.8 | 6.93333 |
| Peyrusse Vieille, EMEP | 43.630272 | 0.17972 |
| Plateliai | 56.01 | 21.88695 |
| Praha, Libus, EMEP | 50.007313 | 14.44620 |
| Prebuz | 50.372444 | 12.61536 |
| Primda, Sumava | 49.66959 | 12.67785 |
| Rao, EMEP | 57.393665 | 11.91417 |
| Rucava, EMEP | 56.161957 | 21.17322 |
| Rudolice v Horach, Krusne hory | 50.57979 | 13.41922 |
| Rychory, Krkonose | 50.66046 | 15.85006 |
| Sedlonov, Polom | 50.3505 | 16.32270 |
| Serlich, Orlicke hory | 50.32804 | 16.38353 |
| Sonnblick, EMEP | 47.05403 | 12.95766 |
| Spitsbergen, Zeppelinfjell, EMEP | 78.88 | 11.88333 |
| Stitna nad Vlari-Popov, Planavy | 49.04776 | 18.00781 |
| Storhofdi, EMEP | 63.4 | -20.28333 |
| Svratouch, EMEP | 49.73507 | 16.03413 |
| Trebic, CHMI station | 49.223446 | 15.86578 |
| Ufa, ERPC | 54.46645 | 56.01233 |
| Valasske Mezirici, observatory | 49.46365 | 17.97371 |
| Westerland, EMEP | 54.892 | 8.32500 |
| Zmiinyi Island, EMEP | 45.25611 | 30.20106 |
| Sedlonov, Polom | 50.3505 | 16.32270 |
| Serlich, Orlicke hory | 50.32804 | 16.38353 |
| Sonnblick, EMEP | 47.05403 | 12.95766 |
| Spitsbergen, Zeppelinfjell, EMEP | 78.88 | 11.88333 |
| Stitna nad Vlari-Popov, Planavy | 49.04776 | 18.00781 |
| Storhofdi, EMEP | 63.4 | -20.28333 |
| Svratouch, EMEP | 49.73507 | 16.03413 |
| Trebic, CHMI station | 49.223446 | 15.86578 |
| Ufa, ERPC | 54.46645 | 56.01233 |
| Valasske Mezirici, observatory | 49.46365 | 17.97371 |
| Westerland, EMEP | 54.892 | 8.32500 |
| Zmiinyi Island, EMEP | 45.25611 | 30.20106 |

Table SI-2. List of freshwater monitoring sites in Slovakia, Czech Republic, The Netherlands, Norway and Russia at which silicone rubber samplers were deployed.

| River/water body | Detailed location ID | Latitude | Longitude |
| --- | --- | --- | --- |
| Danube | Cunovo, static exposure | 48.028905 | 17.220458 |
| Danube | Cunovo, static exposure | 48.028905 | 17.220458 |
| Danube | Cunovo, static cage | 48.028905 | 17.220458 |
| Morava | Bělov Morava | 49.218099 | 17.501749 |
| Morava | Spytihněv Morava | 49.135656 | 17.50277 |
| Morava | Malenovice Dřevnice | 49.207819 | 17.554618 |
| Morava | CR - Čerťák rameno | 49.068912 | 17.435433 |
| Morava | CE - Čerťák Morava | 49.067325 | 17.436385 |
| Svratka |  | 48.961106 | 16.542879 |
| Svratka |  | 48.949673 | 16.550343 |
| Svitava |  | 49.124866 | 16.628388 |
| Svitava |  | 49.093958 | 16.619314 |
| Meuse | s'Hertogenbosh | 51.774313 | 5.346494 |
| Meuse | Maren Kessel | 51.80307 | 5.379518 |
| Meuse | Maren Kessel | 51.81089 | 5.40999 |
| Meuse | Rotterdam | 51.89778 | 4.30240 |
| Meuse | Stevensweert | 51.132880 | 5.843244 |
| Roer | Meuse tributary | 51.14821 | 6.03636 |
| Niers | Meuse tributary | 51.714276 | 5.959418 |
| Groote Molenbeek |  | 51.52797 | 6.07893 |
| Lake Ketelmeer | Lelystad | 52.60896 | 5.65542 |
| lake Ketelmeer | Middle | 52.59934 | 5.762656 |
| Lake Ketelmeer |  | 52.60896 | 5.65542 |
| lake Ketelmeer |  | 52.59934 | 5.762656 |
| Vossenmeer | near Ketelmeer | 52.579504 | 5.775452 |
| Hollandsdiep |  | 51.69262 | 4.48597 |
| Grevelingen |  | 51.73317 | 3.97577 |
| Westerschelde |  | 51.449935 | 4.013381 |
| Eefde |  | 52.15986 | 6.23636 |
| Wolderwijd |  | 52.33172 | 5.56171 |
| Amsterdam dock |  | 52,37917 | 4.93884 |
| Apeldoorns canal |  | 52.439560 | 6.073982 |
| Baakse brook |  | 52.088811 | 6.221503 |
| Afleiding canal |  | 52.157547 | 6.242872 |
| Flevopolder Lagevaart 1 |  | 52.575131 | 5.75178 |
| Graafse Raam |  | 51.768557 | 5.730732 |
| Grote Wetering |  | 51.823304 | 5.432233 |
| Hertogswetering |  | 51753937 | 5.355580 |
| IJssel Zwolle 1 |  | 52.551573 | 5.925392 |
| Meppelerdiep |  | 52.644501 | 6.084168 |
| Overijsselse Vecht |  | 52.518112 | 6.212735 |
| Soesterwetering Zwolle |  | 52.515358 | 6.086384 |
| Alna |  | 59.904628 | 10.791605 |
| Drammen |  | 59.753047 | 10.007518 |
| Glomma |  | 59.306399 | 11.135912 |
| Borgebekken | Borgekrysset | 59.2389921 | 10.2705625 |
|  | Borgeskogen øst | 59.2387633 | 10.2661768 |
|  | Borgeskogen Nord | 59.2539512 | 10.2642077 |
|  | Tarrandrødbekken | 59.290071 | 10.311777 |
| Sandeelva |  | 59.579844 | 10.21848 |
| Toknesbekken |  | 59.175465 | 10.378786 |
| Grennesbekken |  | 59.505097 | 10.097663 |
| Herlandselva |  | 59.41469 | 9.974587 |
| Lysakerelva |  | 59.914108 | 10.635204 |
| Randselva | Site 1 | 60.20529 | 10.34216 |
|  | Site 2 | 60.200531 | 10.337131 |
|  | Site 3 | 60.18797 | 10.29781 |
| Tana |  | 70.39284 | 28.21453 |
| Neiden |  | 69.68741 | 29.39022 |
| Pasvik | Nedre | 69.50112305 | 30.11545385 |
|  | Øvre | 69.42343522 | 29.87864264 |
|  |  | 69.344479 | 30.044867 |
| Kola |  | 68.85018 | 33.02924 |
| Grense Jakobselv |  | 69.76232 | 30.850493 |
| Pechenga |  | 69.53889 | 31.19548 |
| Titovka |  | 69.480273 | 31.829061 |
| Lake Salmijarvi |  | 69.43562 | 30.1261 |
|  |  | 69.43582 | 30.12613 |
| Bayelva |  | 78.93256 | 11.83498 |

Table SI-3. List of monitoring stretches of the Danube sampled with silicone rubber samplers exposed using the dynamic passive sampler exposure unit during the Joint Danube Survey 3[[2]](#footnote-3).

| River/water body | Detailed location ID | Latitude (start) | Longitude (start) | Latitude (end) | Longitude (end) |
| --- | --- | --- | --- | --- | --- |
| Danube | Passau-Bratislava | 48.57851 | 13.408638 | 48.031601 | 17.2219 |
|  | Bratislava-Budapest | 48.031601 | 17.2219 | 47.499594 | 19.043502 |
|  | Budapest-Vukovar | 47.499594 | 19.043502 | 45.365742 | 18.992466 |
|  | Vukovar-Belgrade | 45.365742 | 18.992466 | 44.835712 | 20.448955 |
|  | Belgrade-Turnu-Severin | 44.835712 | 20.448955 | 44.616746 | 22.656572 |
|  | Turnu-Severin-Ruse | 44.616746 | 22.656572 | 43.846114 | 25.935894 |
|  | Ruse-Braila | 43.846114 | 25.935894 | 45.240408 | 27.965033 |
|  | Braila-Tulcea | 45.240408 | 27.965033 | 45.183083 | 28.795907 |

Table SI-4. List of marine monitoring sites at which silicone rubber samplers were deployed.

| Marine location | Detailed sampling location | Latitude | Longitude |
| --- | --- | --- | --- |
| Svalbard | Kongsfjord | 78.96584 | 11.86499 |
| Svalbard | Kvadehuken | 78.9712 | 11.29884 |
| Svalbard | Gåsebu | 78.91236 | 12.09751 |
| Skaggerak | Hvaler | 59.09655 | 11.05073 |
| Oslofjord | Gåsøya | 59.85527 | 10.59527 |
| Norwegian Sea | Ålesund | 62.46322 | 6.22077 |
| Skaggerak | Kristiansand - Glencore | 58.136882 | 7.97644 |
| Skaggerak | Kristiansand - Svensholmen | 58.125304 | 7.985665 |
| Norwegian Sea | Andøya | 69.257367 | 16.17285 |
| Barents Sea - Bear Island | Bear Island | 74.512 | 18.966167 |
| Greenland Sea - Jan Mayen | | 71.09445 | -8.897182 |
| Inner Oslofjord | Akershus festning | 59.904234 | 10.728579 |
| Aegean Sea | Middle bay | 35.346874 | 25.69806 |
| Aegean Sea | Taverna bay | 35.312033 | 25.437445 |
| Aegean Sea | Thessaloniki | 40.510185 | 22.702087 |
| Aegean Sea | Thessaloniki | 40.50113 | 22.800055 |
| Aegean Sea | Thessaloniki | 40.603793 | 22.90097 |
| Black Sea | Ukraine Site 1a | 46.49342 | 30.775861 |
| Black Sea | Ukraine Site 2a | 45.254573 | 30.206683 |
| Atlantic Coast (Portugal) | Rio Formosa lagoon | 37.030758 | -7.796766 |
|  | Rio Formosa lagoon | 37.01423 | -7.851934 |

Table SI-5. List of monitoring stretches of the Black Sea sampled with silicone rubber samplers exposed using the dynamic passive sampler exposure unit.

| River/water body | Detailed location ID | Latitude (start) | Longitude (start) | Latitude (end) | Longitude (end) |
| --- | --- | --- | --- | --- | --- |
| Black Sea | Stretch 1 | 46.503466 | 30.749695 | 46.503466 | 30.749695 |
|  | Stretch 2 | 46.503466 | 30.749695 | 41.664203 | 41.640898 |
|  | Stretch 3 | 41.664203 | 41.640898 | 41.664203 | 41.640898 |
|  | Stretch 4 | 41.664203 | 41.640898 | 44.116267 | 28.687016 |

**2. Henry’s law constants for HCB and PeCB**

In general, no substantial differences in Henry’s law constants have been reported for HCB and PeCB (Table SI-6). Based on H values, we should not expect large differences in ratios of HCB and PeCB concentrations in air and water when close to equilibrium. Slopes of change in the natural logarithm of H with temperature are also relatively similar indicating that temperature will not profoundly affect the ratio of H values for HCB and PeCB (Figure SI-1).

Table SI-6. Summary of literature values of Henry’s law constants for HCB and PeCB.

| **Henry’s law constant**  **(at 20 °C)** | | ** ΔlnH/Δ(1/T)** | | **H_PeCB_/H_HCB_** | **Reference** |
| --- | --- | --- | --- | --- | --- |
| HCB | PeCB | HCB | PeCB |  |  |
| 0.015 | 0.014 | 6000 | 5200 | 0.93 | Shen and Wania (2005) |
| 0.019 | 0.014 | 6400 | 5600 | 0.74 |  |
| 0.03 |  |  |  |  | Jantunen and Bidleman (2006) |
| 0.02 | 0.014 | 5700 | 5200 |  | Ten Hulsher et al. (1992) |
| 0.0076 | 0.012 | 6400 | 5700 | 1.58 | Mackay and Shiu (1981) |
|  |  |  |  |  |  |

Figure SI-1. Change in the ratio of Henry’s law constants for PeCB and HCB (H_PeCB_/H_HCB_) with temperature.

**3. Ratio of HCB and PeCB in the Drammen and Glomma rivers**

The following figure shows how the HCB/PeCB ratios estimated for these two rivers varies with sampling rate R_s_, degree of equilibrium achieved (DEq), and the average deployment temperature (Figure SI-2). We attempted to evaluate the spread in the HCB/PeCB ratios observed for the Drammen and Glomma rivers by comparing these with the ratios obtained by randomly varying the C_w_ (n= 22) for HCB and PeCB by either +/- 15 or 30 % around two concentration values resulting in a HCB/PeCB ratio of 4.1 (Figure SI-3).

Figure SI-2. Ratio of freely dissolved aqueous HCB and PeCB concentrations plotted as a function of sampling rate, R_s_ at logK_sw_ = 5 (L d^-1^), degree of equilibrium (DEq at logK_sw_ = 4.5), and average water temperature for silicone rubber passive samplers deployed consecutively over a 3-year period in the Drammen and Glomma rivers (Norway).

Figure SI-3. Boxplot of HCB/PeCB ratios estimated for the Drammen (n = 22) and Glomma (n = 17) rivers with modelled range of ratios generated based on randomly generated ratios with variability of 15 and 30 % around the mean value of 4.2 (n = 22).

**4. Proportion of HCB and PeCB dissolved and sorbed to SPM and DOC in the Drammen and Glomma rivers.**

Regular monitoring of HCB in the dissolved and suspended particulate matter was conducted from 2013-2016 alongisde with measurements of supporting parameters such as dissolved organic carbon and SPM content of the water ([DOC] and [SPM]), organic carbon content of the SPM. These data are summarized in the table below and used to estimate the proportion of freely dissolved HCB present or associated with SPM and DOC (with logK_DOC_ from Burkhard, 2000 ). In general, HCB appears to be primarily found in the dissolved form in these two rivers. About 80-90 % of HCB is freely dissolved while 3 and 7 % on average is bound to SPM and DOC. Considering that PeCB is less hydrophobic than HCB, a larger proportion will be present as dissolved form.

Table SI-7. Summary of HCB concentration data and supporting parameters from routine monitoring of the Drammen and Glomma rivers (2013-2016).

| **Parameter** | **HCB** | **Reference** |
| --- | --- | --- |
| [SPM] (mg L^-1^) | 1.6-55 |  |
| % OC (ng g^-1^) | 2-11 |  |
| C_SPM_ (ng g^-1^) | <0.3-1 |  |
| [DOC] (mg L^-1^) | 3-3.6 |  |
| C_w_ (ng L^-1^) | 0.015-0.4 | Skarbovik et al. (2017) |
| logK_DOC_ (L kg^-1^) | 4.4 | Burkhard, (2000) |
| Dissolved fraction (%) | 89 (81-91) |  |
| Fraction sorbed to SPM (%) | 3 (2.9-11) |  |
| Fraction sorbed to DOC (%) | 7 (6-8) |  |

**5. Relative percent differences in calculated freely dissolved concentrations from duplicate passive sampling measurements**

Relative percent difference (% RPD) was calculated for C_w_ from 79 pairs of duplicate SR samplers deployed alongside and analyzed at NIVA. The % RPD of the resulting HCB/PeCB ratios are also displayed. The median of % RPDs for calculated C_w_ for HCB and PeCB is just above 10 % in both cases. The median of % RPD of the ratio is lower and approximately 5 %. This indicates that the use of duplicate deployments of SR passive samplers does not significantly increase the robustness of the measurement compared with a single deployment.

Figure SI-4. Relative percent differences (% RPD) in calculated freely dissolved concentrations (C_w_) of PeCB and HCB and HCB/PeCB ratios for replicate silicone rubber passive sampling measurements (n = 79).

**6. HCB/PeCB concentration ratios at freshwater sites**

The boxplot below presents the HCB/PeCB concentration ratio found at all freshwater sites of interest.

Figure SI-5. HCB/PeCB ratio of freely dissolved concentrations estimated from silicone rubber passive samplers deployed in freshwater environments ordered by latitude (north) from 43.7 (Danube) to 78.9 (Bayelva) during the period 2010-2019. Rivers shown in blue are for environments for which concentrations of both compounds are expected not to be affected by point source contamination. Freshwater environments for which impact from possible local emission sources of PeCB and/or HCB is unknown are shown in dark orange. Note that the number of measurements n per box is different for the different marine environments (see Table SI-10).

**6. HCB/PeCB concentration ratios at marine sampling sites**

The boxplot below presents the HCB/PeCB concentration ratios found at all marine sampling sites of interest.

Figure SI-6. HCB/PeCB ratio of freely dissolved concentrations estimated from silicone rubber passive samplers deployed in marine or coastal environments between 35 and 78.9 latitude north during the period 2010-2017. Sampling locations shown in blue are for environments for which concentrations of both compounds are expected not to be affected by point source contamination. Marine environments for which impact from possible local emission sources of PeCB and/or HCB is unknown are shown in dark orange. Note that the number of measurements n per box is different for the different marine environments (see Table SI-10).

**8. Freely dissolved concentrations, C_w_ for PeCB and HCB estimated at the different monitoring locations**

The graphs below present a comparison of estimated PeCB and HCB concentrations from SR sampling at different freshwater or marine sites. Concentrations were calculated according to the procedure given in the main text. No corrections were applied for differences in water temperature and salinity at the different sites.

Figure SI-7. Relationship between freely dissolved concentrations of HCB and PeCB estimated from SR passive sampling conducted at freshwater and marine sites from 2010 to 2019. See the main text for the statistics

**7. Benchmarking of CB congeners 28 and 52 with PeCB and HCB**

Boxplots presenting benchmarking of levels of PCB congeners 28 and 52 with those of PeCB and HCB are shown below in Figures SI-8 and SI-9 for marine and freshwater environments, respectively.

Figure SI-8. Benchmarking of freely dissolved concentrations of PCB congeners 28 and 52 estimated from silicone rubber passive samplers deployed in the marine environments between 35 and 78.9 latitude north during the period 2010-2019 with concentrations of HCB and PeCB from the same samplers. Sampling locations shown in blue are for environments for which concentrations of both compounds are expected not to be affected by point source contamination. Marine environments for which impact from possible local emission sources of PCBs is unknown are shown in dark orange. Note that the number of measurements n per box is different for the different marine environments (see Table SI-10).

Figure SI-9. Benchmarking of freely dissolved concentrations of PCB congeners 28 and 52 estimated from silicone rubber passive samplers deployed in freshwater environments between 35 and 78.9 latitude north during the period 2010-2019 with concentrations of HCB and PeCB from the same samplers. Sampling locations shown in blue are for environments for which concentrations of both compounds are expected not to be affected by point source contamination. Freshwater environments for which impact from possible local emission sources of PCBs are shown in dark orange. Note that the number of measurements n per box is different for the different marine environments (see Table SI-9).

**9. Levels of PCBs in cod along the Norwegian coast**

Regular monitoring of PCBs on the Norwegian coast is generally undertaken using Atlantic cod (*Gadus morhua*). These data also form part of the data that is submitted yearly to OSPAR. Here we have chosen cod data from three relevant sites (namely Hvaler, Oslofjord and Ålesund) for which corresponding passive sampling data was available for the period 2012-2016. Cod data were obtained from the national database on contaminants in the Norwegian environment (<https://vannmiljo.miljodirektoratet.no/>). Figure SI-10 shows that levels of PCBs in the inner Oslofjord are higher than the two other sites along the coast. This is the same assessment we were able to undertake with benchmarking ratios of Figure SI-8 or intrinsic freely dissolved concentrations of Figure SI-10. The benchmarking on Figure SI-8 shows, as below, that PCBs concentrations tend to be higher at the Ålesund sampling site than in Hvaler.

Figure SI-10. Sum of lipid-normalised concentration (ng g^-1^ lip) of 7 indicator PCBs (CB congeners 28, 52, 101, 118, 138, 153 and 180) in cod liver (*Gadus morhua*) sampled at three locations along the Norwegian coast and reported during the period 2012-2016. These locations correspond to the locations where passive sampling was undertaken during the same period.

**10. Freely dissolved concentrations, C_w_ for PCB28 and PCB52 estimated at the different monitoring locations**

The boxplots below present a comparison of estimated PCB28 and PCB52 concentrations from SR sampling at different freshwater or marine sites. Concentrations were calculated according to the procedure given in the main text. No corrections were applied for differences in water temperature and salinity at the different sites.

Figure SI-11. Freely dissolved concentrations of PCB congeners 28 and 52 estimated from silicone rubber passive samplers deployed in the freshwater environments between 35 and 78.9 latitude north during the period 2010-2019. Sampling locations shown in blue are for environments for which concentrations of both compounds are expected not to be affected by point source contamination. Freshwater environments for which impact from possible local emission sources of PeCB and/or HCB is unknown are shown in dark orange. Note that the number of measurements n per box is different for the different freshwater environments (see Table SI-9).

Figure SI-12. Freely dissolved concentrations of PCB congeners 28 and 52 estimated from silicone rubber passive samplers deployed in the marine environments between 35 and 78.9 latitude north during the period 2010-2019. Sampling locations shown in blue are for environments for which concentrations of both compounds are expected not to be affected by point source contamination. Marine environments for which impact from possible local emission sources of PeCB and/or HCB is unknown are shown in dark orange. Note that the number of measurements n per box is different for the different marine environments (see Table SI-10).

**11. Freely dissolved concentrations in water (data)**

Concentrations of HCB, PeCB, CB28 and CB52 are given in ng L^-1^ in Table SI-8 below. Grey shaded cells indicate data below LOQ and reported LOQ.

Table SI-8. Summary of individual passive sampler deployments.

Table SI-9. Number of samples (n) for each freshwater sampling locations used in the boxplots in SI.

| River/water body | n |
| --- | --- |
| Danube | 11 |
| Morava | 5 |
| Svratka | 2 |
| Svitava | 2 |
| Meuse | 8 |
| Roer | 1 |
| Niers | 1 |
| Groote Molenbeek | 1 |
| Lake Ketelmeer | 4 |
| Vossenmeer | 1 |
| Hollandsdiep | 2 |
| Grevelingen | 1 |
| Westerschelde | 1 |
| Eefde | 1 |
| Wolderwijd | 1 |
| Amsterdam dock | 1 |
| Apeldoorns canal | 1 |
| Baakse brook | 1 |
| Afleiding canal | 1 |
| Flevopolder Lagevaart 1 | 1 |
| Graafse Raam | 1 |
| Grote Wetering | 1 |
| Hertogswetering | 1 |
| IJssel Zwolle 1 | 1 |
| Meppelerdiep | 1 |
| Overijsselse Vecht | 1 |
| Soesterwetering Zwolle | 1 |
| Alna | 26 |
| Drammen | 22 |
| Glomma | 17 |
| Borgebekken | 5 |
| Sandeelva | 1 |
| Toknesbekken | 1 |
| Grennesbekken | 1 |
| Herlandselva | 1 |
| Lysakerelva | 2 |
| Randselva | 3 |
| Tana | 3 |
| Neiden | 2 |
| Pasvik | 12 |
| Kola | 4 |
| Grense Jakobselv | 6 |
| Pechenga | 4 |
| Titovka | 2 |
| Lake Salmijarvi | 4 |
| Bayelva | 2 |

Table SI-10. Number of samples (n) for each marine sampling locations used in the boxplots in SI.

| Marine location |  |  |
| --- | --- | --- |
| Svalbard | Kongsfjord | 3 |
| Svalbard | Kvadehuken | 2 |
| Svalbard | Gåsebu | 3 |
| Skaggerak | Hvaler | 8 |
| Oslofjord | Gåsøya | 8 |
| Norwegian Sea | Ålesund | 8 |
| Skaggerak | Kristiansand - Glencore | 6 |
| Skaggerak | Kristiansand - Svensholmen | 6 |
| Norwegian Sea | Andøya | 2 |
| Barents Sea - Bear Island | Bear Island | 4 |
| Greenland Sea | Jan Mayen | 2 |
| Inner Oslofjord | Akershus festning | 2 |
| Aegean Sea | Middle bay | 5 |
| Black Sea |  | 6 |
| Atlantic Coast | Lagoon | 6 |

**12. References**

1. Shen L & Wania F (2005) Compilation, evaluation, and selection of physical− chemical property data for organochlorine pesticides. *Journal of Chemical & Engineering Data* 50(3):742-768.

2. Jantunen LM & Bidleman TF (2006) Henry’s law constants for hexachlorobenzene, p, p′-DDE and components of technical chlordane and estimates of gas exchange for Lake Ontario. *Chemosphere* 62(10):1689-1696.

3. Hulscher TET, Van Der Velde L, & Bruggeman W (1992) Temperature dependence of Henry's law constants for selected chlorobenzenes, polychlorinated biphenyls and polycyclic aromatic hydrocarbons. *Environmental Toxicology and Chemistry: An International Journal* 11(11):1595-1603.

4. Mackay D & Shiu WY (1981) A critical review of Henry’s law constants for chemicals of environmental interest. *Journal of physical and chemical reference data* 10(4):1175-1199.

5. Burkhard LP (2000) Estimating dissolved organic carbon partition coefficients for nonionic organic chemicals. *Environmental Science & Technology* 34(22):4663-4668.

6. Skarbøvik E*, et al.* (2015) Riverine Inputs and Direct Discharges to Norwegian Coastal Waters-2014.

1. <https://data.genasis.cz/> [↑](#footnote-ref-2)
2. <http://www.danubesurvey.org/jds3/results> [↑](#footnote-ref-3)
